# Supplementary material for: Prevalence and Demographic, Socioeconomic, and Behavioral Risk Factors of Self-Reported Symptoms of Sexually Transmitted Infections (STIs) among Ever-Married Women: Evidence from Nationally Representative Surveys in Bangladesh
Source: Int J Environ Res Public Health. 2022 Feb 8;19(3):1906. doi: 10.3390/ijerph19031906 (PMC8835257; doi:10.3390/ijerph19031906)
Supplement: Supplementary file 1 [file ijerph-19-01906-s001.zip › ijerph-1579945-supplementary.pdf]

Supplementary Table S1. Logistic regression on the association of abnormal genital discharge and genital sores with demographic, socio-economic and behavioural risk factors (multiple imputation analysis).

Study factors	Model 3 (abnormal genital discharge)	95% CI	*p*-value	Model 3 (genital sores)	95% CI	*p*-value
Demographic factors						
Age (Ref:15-24 years)						
25-34 years	1.14**	(1.04 - 1.26)	0.008	1.19**	(1.06 - 1.35)	0.004
35-49 years	0.92	(0.83 - 1.02)	0.114	1.02	(0.89 - 1.17)	0.813
Age at first marriage (Ref: 18 years or above)						
9-14 years	1.10	(0.98 - 1.24)	0.106	1.04	(0.91 - 1.18)	0.569
15-17 years	1.18*	(1.05 - 1.32)	0.004	1.05	(0.92 - 1.20)	0.470
Type of residence (Ref: Rural)						
Urban	1.01	(0.91 - 1.13)	0.827	1.07	(0.94 - 1.22)	0.277
Socio-economic factors						
Education (Ref: No education)						
Primary	1.08	(0.95 - 1.22)	0.220	1.13	(0.99 - 1.29)	0.078
Secondary	0.95	(0.83 - 1.09)	0.479	1.09	(0.93 - 1.28)	0.293
Higher	0.78*	(0.62 - 0.98)	0.032	1.06	(0.81 - 1.40)	0.655
Partner's education (Ref: No education)						
Primary	1.06	(0.96 - 1.18)	0.242	1.05	(0.92 - 1.19)	0.481
Secondary	0.97	(0.87 - 1.09)	0.638	0.99	(0.86 - 1.14)	0.880
Higher	0.87	(0.73 - 1.03)	0.100	0.90	(0.73 - 1.12)	0.344
Wealth quintile (Ref: Poorest)						
Poorer	1.02	(0.91 - 1.15)	0.728	1.01	(0.87 - 1.17)	0.938
Middle	0.95	(0.83 - 1.08)	0.436	0.89	(0.76 - 1.05)	0.168
Richer	0.88	(0.77 - 1.01)	0.079	0.86	(0.72 - 1.03)	0.109
Richest	0.73***	(0.61 - 0.86)	<0.001	0.76**	(0.61 - 0.93)	0.008
Paid work status (Ref: No)						
Yes	1.13*	(1.02 - 1.26)	0.019	1.20**	(1.06 - 1.35)	0.003
Behavioral factors						
Contraceptive method (Ref: No method)						
Traditional method	1.40***	(1.22 - 1.61)	<0.001	1.12	(0.94 - 1.33)	0.214
Modern method	1.08	(1.00 - 1.18)	0.063	1.08	(0.98 - 1.20)	0.111
Knowledge about STI (Ref: No)						
Yes	1.10	(0.99 - 1.23)	0.081	1.27***	(1.13 - 1.43)	<0.001
Wife beating justified (Ref: No)						
Yes	1.01	(0.89 - 1.16)	0.834	1.29*	(1.11 - 1.51)	0.001
Women's healthcare decision-making (Ref: Wife)						
Wife and husband	0.77	(0.69 - 0.87)	<0.001	0.81*	(0.70 - 0.93)	0.002
Respondent and someone else	0.87	(0.73 - 1.04)	0.137	0.78*	(0.62 - 0.97)	0.025
Husband	0.93	(0.81 - 1.05)	0.246	0.87	(0.76 - 1.01)	0.062

Exposure to mass media (Ref: Not at all)						
Irregular	1.22*	(1.07 - 1.38)	0.002	1.01	(0.85 - 1.18)	0.950
Regular	1.11*	(1.00 - 1.22)	0.047	1.02	(0.90 - 1.16)	0.737
Survey year (Ref: 2007)						
Survey Year = 2011	1.48***	(1.31 - 1.68)	<0.001	1.39***	(1.20 - 1.60)	<0.001
Survey Year = 2014	1.54***	(1.35 - 1.75)	<0.001	1.04	(0.90 - 1.21)	0.572
Constant	0.07***	(0.06 - 0.09)	<0.001	0.04***	(0.03 - 0.06)	<0.001
Observation		43,557			43,557	

Note: *** p<0.001, ** p<0.01, * p<0.05
